# Role of autophagy in aging: The good, the bad, and the ugly

**DOI:** 10.1111/acel.13753

**Published:** 2022-12-20

**Authors:** Siamak Tabibzadeh

**Affiliations:** ^1^ Frontiers in Bioscience Research Institute in Aging and Cancer Irvine California USA

**Keywords:** aging, AMPK, cancer, mTOR, SASP, senescence

## Abstract

Autophagy (self‐eating) is a conserved catabolic homeostatic process required for cellular metabolic demands by removal of the damaged molecules and organelles and for alleviation of stress initiated by pathology and infection. By such actions, autophagy is essential for the prevention of aging, disease, and cancer. Genetic defects of autophagy genes lead to a host of developmental, metabolic, and pathological aberrations. Similarly, the age‐induced decline in autophagy leads to the loss of cellular homeostatic control. Paradoxically, such a valuable mechanism is hijacked by diseases, during tumor progression and by senescence, presumably due to high levels of metabolic demand. Here, we review both the role of autophagy in preventing cellular decline in aging by fulfillment of cellular bioenergetic demands and its contribution to the maintenance of the senescent state and SASP by acting on energy and nutritional sensors and diverse signaling pathways.

## Graphical abstract

Various forms of autophagy. Major types of autophagy include macroautophagy, microautophagy (inside the circle), and chaperone‐mediated autophagy. Autophagy is used for the removal of damaged biomolecules and organelles (outside the circle).

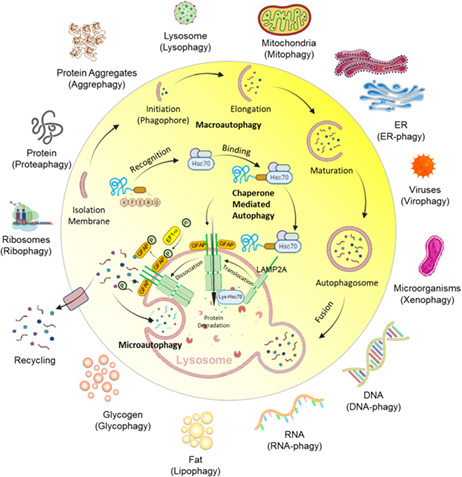

## 1 INTRODUCTION

The term “autophagy” existed from the middle of the 19th century and was coined in 1963 by the Nobel Laurette, Christian René Marie Joseph, Viscount de Duve, after discovery of lysosomes (Porter & Novikoff, 1974). Since then, the intricate nature of the process has come to light. It is shown that autophagy is required for fulfillment of cellular metabolic demands, preservation of genomic integrity, innate, and adaptive immune processes, regulation of pro‐inflammatory mediators and for forstering the cell survival. Autophagy is evolutionarily conserved and serves as a ubiquitous, self‐degradative catabolic process that removes long lived, damaged molecules and organelles, aggregated proteins, and intracellular pathogens.

## 2 DIVERSE FORMS OF AUTOPHAGY

**FIGURE 1:**
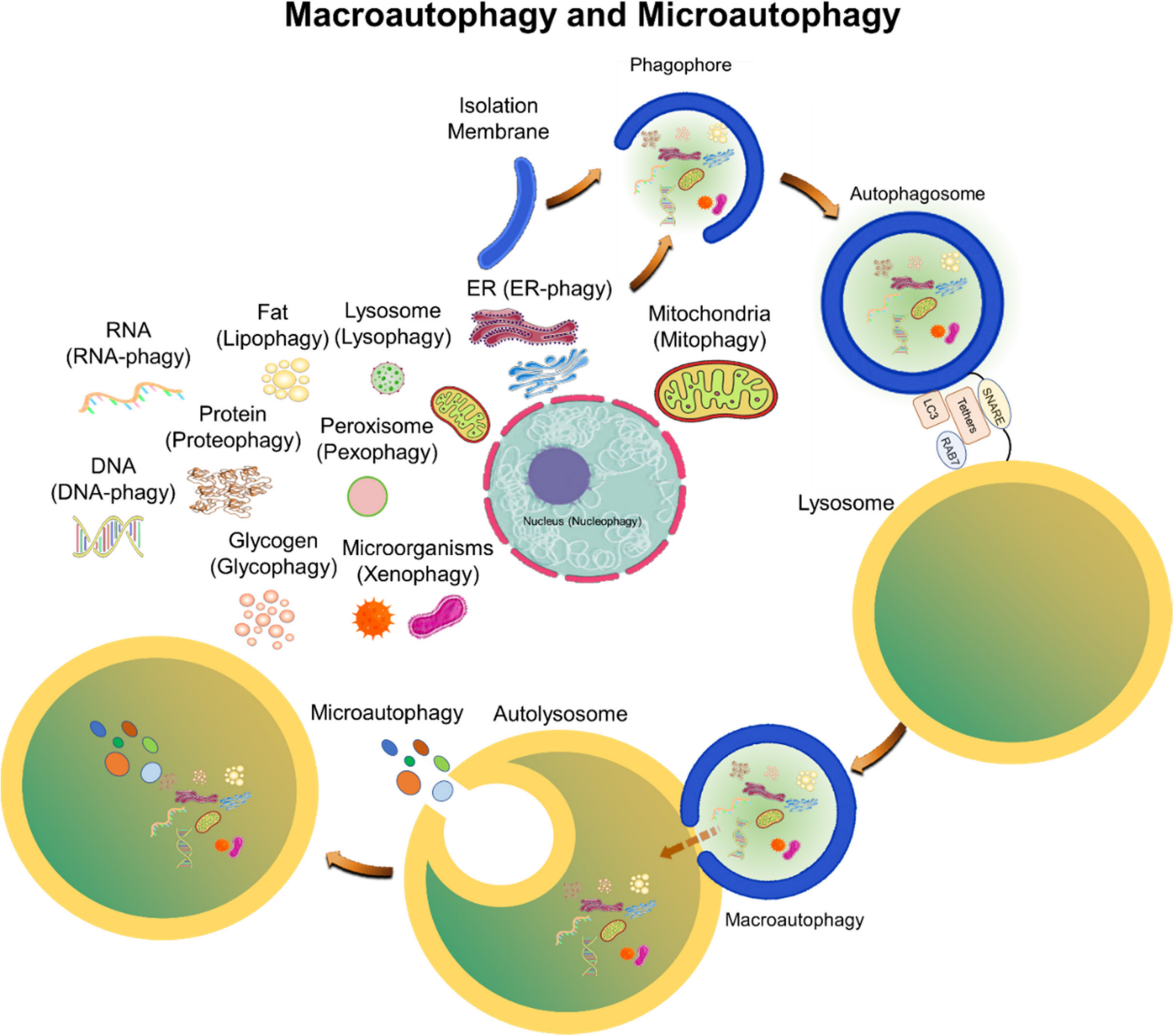
Schematic depiction of macroautophagy and microautophagy. In macroautophagy, random dysfunctional organelles and damaged molecules are engulfed by the elongating phagophore. Phagophore is ultimately sealed, forming a double‐membrane‐bound autophagosome that, by virtue of tethers, binds, and then fuses with lysosome. This creates the autolysosome, where the transferred cargo, is degraded by autosomal hydrolases. In microautophagy, cargoes are directly sequestered by the invagination of the vacuolar membrane. This is then followed by scission, subsequent lysis of membrane, exposing cargo to vacuolar hydrolases for degradation (Schütter et al. 2020 and Aman et al., 2021, Yim and Mizushima 2020). Produced at biorender.com.

Basal autophagy occurs constitutively in nutrient‐rich environments for the turnover of the cellular components as a fundamental survival mechanism. However, the induced or reactive autophagy is engaged by stresses such as starvation, low level of amino acids, trophic factor or hormone deprivation, heat, and ER stress, hypoxia, irradiation, drugs, and intracellular pathogens (Komatsu et al., 2007). Major types of autophagy include macroautophagy, microautophagy, and chaperone‐mediated autophagy (CMA). Autophagy is also used for the removal of damaged organelles including mitochondria (mitophagy), ER (ER‐phagy), lysosome (lysophagy), among others and for the removal of damaged macromolecules and foreign pathogens (Figure 1) (Jia et al., 2020).

**FIGURE 2:**
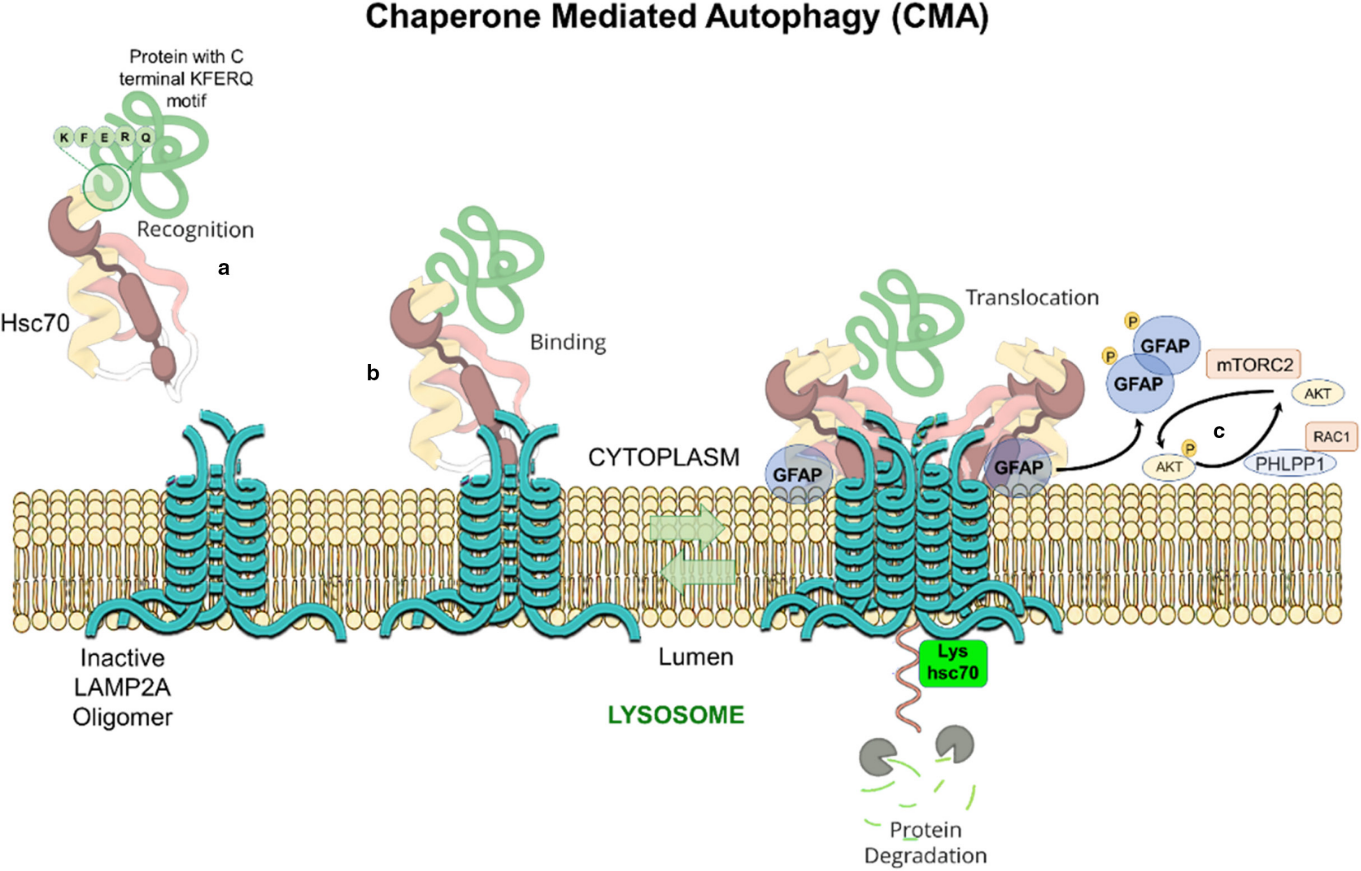
Principal events in chaperone‐mediated autophagy (CMA). (a) CMA protein substrates, destined to be degraded, bind by their cytoplasmic C‐terminal KFERQ motifs, to the heat shock cognate 71 kDa protein (Hsc70). (b) CMA protein substrates and Hsc70 can bind to the C‐terminal tail of LAMP2A, an inactive oligomer, that resides in the lysosomal membranes, with a similar affinity. The short C‐terminal tail of LAMP‐2A, comprised of 12 amino acids, is exposed to the cytoplasm and probably binds to the chaperone and the substrate in a competitive manner. (c) High molecular weight LAMP2A aggregates are formed by the participation of glial fibrillary acidic protein (GFAP), creating supramolecular complexes that enter the lysosomal lumen. The signaling pathway, that regulates the stabilization of the LAMP‐2A complex at lysosomal membranes, undergoes both phosphorylating and dephosphorylating signals, imposed by AKT, mTORC2, PHLPP1, RAC1, and GFAP. The level of lysosome‐associated hsc70 (lys‐hsc70) increases proportionally as CMA activity increases and becomes limiting only when LAMP‐2A is in present excess (Alfaro et al., 2019). Image was created at BioRender.com.

Macroautophagy applies for the bulk and non‐selective removal of macromolecules or subcellular organelles within cytosolic double‐membrane‐bound autophagosomes fused with lysosomes (Figure 1) (Mijaljica et al., 2011; Feng et al., 2014; Schütter et al., 2020; Aman et al., 2021; Yim & Mizushima, 2020). Microautophagy and micro‐ERphagy can occur in a non‐selective way for the sequestration of the bulk of cytosolic substrates under starvation conditions or can occur with high precision by allowing specific cargoes to be captured before they enter the invaginations of endolysosome membranes and get digested by lysosomes (Figure 1) (Hansen et al., 2018). Chaperone‐mediated autophagy (CMA), which is induced after long‐term starvation, uses the chaperone, heat shock protein 70 (HSC70; 71‐kDa, also known as HSPA8) for interaction with cytosolic proteins with its C‐terminal KFERQ pentapeptide sequence (Figure 2) (Alfaro et al., 2019, Mizushima et al., 2008). Autophagy uses cargo receptors and uses the interaction with LC3 in autophagic membranes, to specifically remove organelles (Seglen et al., 1990, Zaffagnini and Martens, 2016) (Figure 1).

Protein aggregates are removed by a type of macroautophagy called aggrephagy (Hyttinen et al., 2014, Kaushik and Cuervo 2018). DNA and RNA are bound in an ATP‐dependent and CMA‐like manner (Fujiwara et al., 2015, Aizawa et al., 2017). Glycophagy is a hormonally controlled and highly regulated process, requiring many signaling pathways such as the cyclic AMP protein kinase A/protein kinase A, PI3K‐Akt/PKB‐mTOR, and calcium (Zhao et al., 2018). Lipophagy is required for maintaining the energy balance by the breakdown of lipids. Lipophagy requires the participation of autophagy, by recruiting a complex network of over 32 proteins of autophagy and autophagy‐related proteins. (Greenberg et al., 2011, Fujimoto and Parton, 2011, Singh and Cuervo, 2012). Granulophagy removes the stress granules and P bodies (Buchan et al., 2013). Mitochondrial integrity is maintained and removal of damaged mitochondria becomes feasible in vivo, by the joint participation of two Parkinson's disease genes, mitochondrial kinase, PINK1, and ubiquitin ligase, Parkin. Triggering mitophagy requires PINK1 phosphorylation of Parkin and ubiquitin with USP30 deubiquitinase, which acts as a brake in this process, by opposing Parkin‐mediated ubiquitination. Mitophagy allows damaged mitochondria to be captured by binding the soluble or membrane‐bound mitophagy receptors (mReceptors), followed by binding LC3, recruitment of p62 before engulfment, formation of autophagosomes, and their subsequent delivery to lysosomes for degradation (Onishi et al., 2021, Bingol and Sheng, 2016, Abudu et al., 2021). The term, “MitophAging” is suggested to include age‐induced diseases that result from loss of mitophagy (Bakula and Scheibye‐Knudsen, 2020). Damaged ER fragmentation requires atlastin 2 (ATL2), a GTPase‐mediating homotypic fusion of the ER. Some ER domains, utilize Atg8, whereas certain ER‐specific domains use other types of receptors (Dikic and Elazar, 2018, Stolz and Grumati, 2019).

There is increasing evidence that aging leads to the loss of expression of autophagy genes and substantially reduces the activity of selective as well as non‐selective autophagy (Leidal et al., 2018, Simonsen et al., 2008). Forced genetic impairment of autophagy induces an accelerated decline in cellular functions whereas an increase in autophagy delays aging in animal models (Leidal et al., 2018, Hansen et al., 2018). Aging also results in the generation of oxysterols such as 7‐ketocholesterol‐ and 7β‐hydroxycholesterol. These oxysterols result in the dysfunction of mitochondria and peroxisomes, oxidative stress, and a type of autophagic cell death, called oxiapoptophagy (Nury et al, 2021). Here, we briefly summarize various forms of autophagy.

## 3 SOURCES OF PHAGOPHORE OR ISOLATION MEMBRANE

**FIGURE 3:**
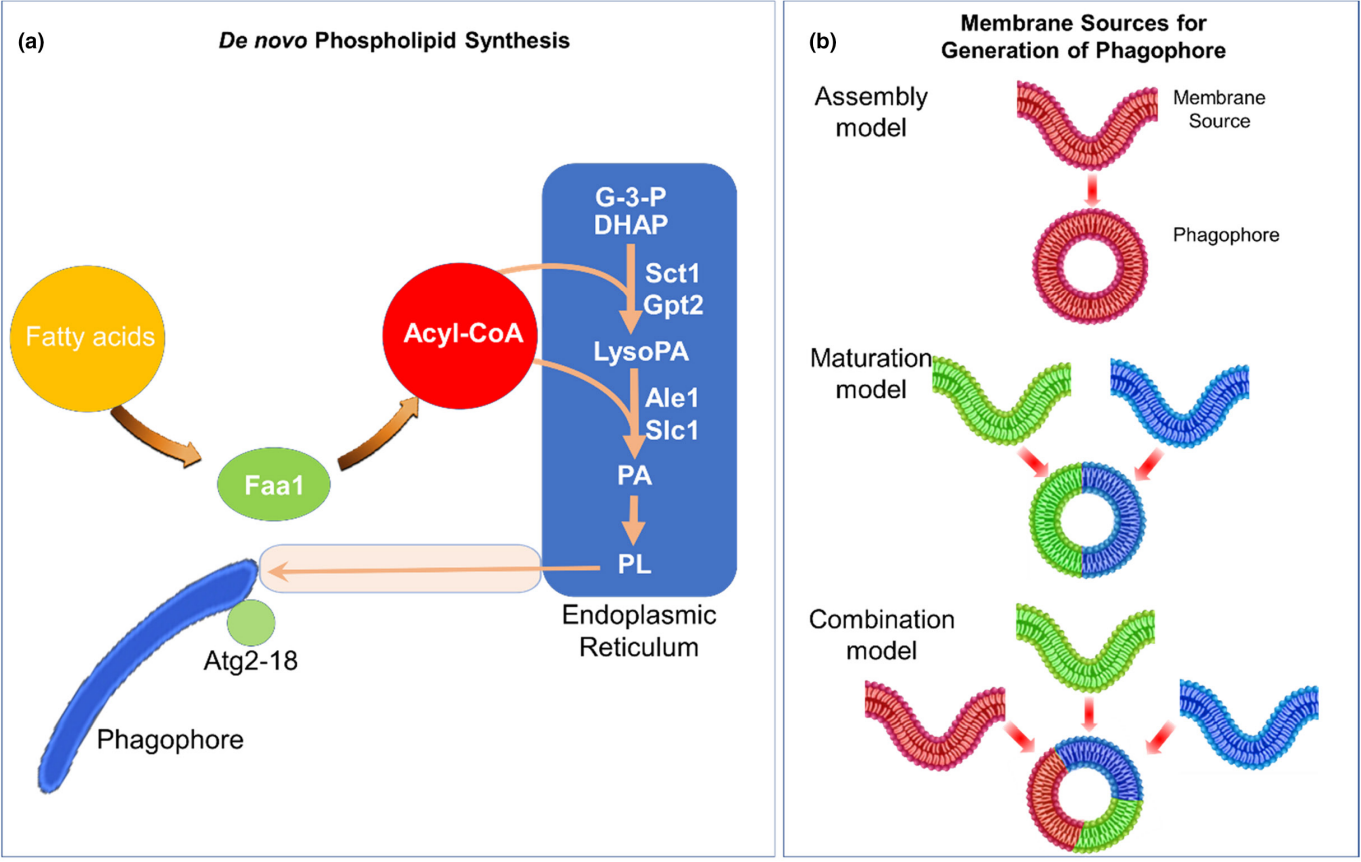
(a) De novo phospholipid synthesis drives phagophore expansion and autophagy. Fatty acyl‐CoA synthetase (Faa1) mediated FA channeling fosters de novo synthesis and transport of phospholipids. This process supports phagophore expansion via the tether and lipid transfer by a complex comprised of Atg2 and Atg18. CoA, coenzyme a; DHAP, dihydroxyacetone phosphate; FA, fatty acids; G‐3‐P, glycerol‐3‐phosphate; PA, phosphatidic acid, PL: Phospholipid (Schütter et al. 2020). (b) Membrane sources for the formation of phagophore. Assembly model: The autophagosome membrane is assembled from different membrane sources. Maturation model: The autophagosome membrane is derived from a pre‐existing cytoplasmic organelle's membrane. Combination model: The autophagosome membrane is derived from a pre‐existing cytoplasmic membrane, and during maturation from other organelles (Bento et al., 2016).

**FIGURE 4:**
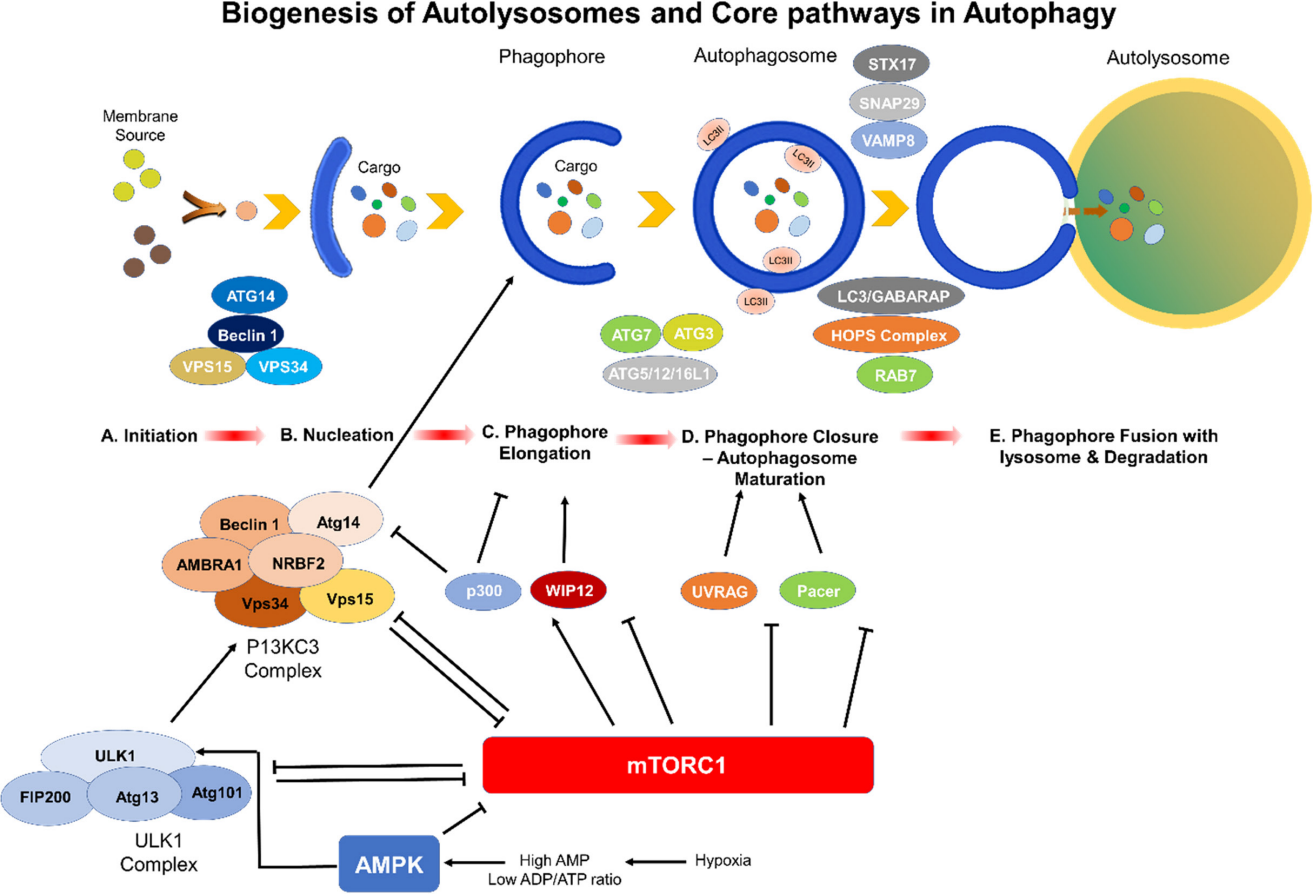
Schematic depiction of the stages of autophagy, and autophagy proteins required for the formation of phagophore, autophagosome, and autolysosome. During this process, the phagophore is elongated and is ultimately closed, forming the autophagosome. Subsequently, the phagosome fuses with the lysosome, allowing the cargo to be degraded with lysosomal hydrolases (Schütter et al. 2020, Aman et al., 2021, Yim and Mizushima 2020). AMPK, which is activated by hypoxia, high AMP or low ADP to ATP ratio, activates ULK1 whereas mTORC1 inhibits ULK1 complex activity by phosphorylating ULK1 and Atg13. mTORC1 inhibits the nucleation phase by phosphorylation of Atg14, AMBRA1, and NRBF2 in the PI3KC3 complex I. mTORC1 inhibits autophagosome elongation by inactivating p300 and WIPI2 through phosphorylation which, in turn, inhibits VSP34 activity, LC3 lipidation, and the recruitment of phosphatidylinositol phosphates as well as LC3 conjugation system. mTORC1 prevents autophagosome‐lysosome fusion by phosphorylation of UVRAG and pacer which are required for the recruitment of HOPS tethering complex and the lipid kinase activity of the P13KC3 complex II (Dossou and Basu, 2019).

During autophagy, small single membrane phagophores, the double‐membrane structures that function in the initial sequestering of cargo, engulf and rapidly expand by localized phospholipid synthesis, and transform into double‐membrane‐bound autophagosomes (Figures 3a and 4) (Schütter et al. 2020, Aman et al., 2021, Yim and Mizushima 2020, Dossou and Basu, 2019). The core proteins that regulate the biogenesis of autophagosomes have been identified, yet, it is not yet quite clear how these structures are assembled. The autophagosomal membranes are either derived from pre‐existing cytoplasmic organelles (maturation model), from different sources (assembly model) or from the combination of both sources (Figure 3b) (Bento et al., 2016). The maturation model is supported by the contacts that are visualized by electron microscopy between autophagosomes and membranes of organelles such as ER, mitochondria, Golgi complex, endosomes, or plasma membranes (Bento et al., 2016). Electron tomography has recently been used to demonstrate that these pre‐existing isolation membranes appear to reside in and form interconnections within a subdomain of the ER. By virtue of dedicated protein tethers, ER network establish membrane contact sites (MSCs) with virtually all cellular organelles (Figure 1) (Schütter et al., 2020).

The biogenesis of the autophagosome is initiated by the formation of conserved autophagy protein machinery at pre‐autophagosomal structures (PASs) which serve as a peri‐vacuolar compartment where the nucleation of the phagophore is initiated (Figure 4). It was recently shown that PAS is formed by the establishment of stable contacts between the endoplasmic reticulum (ER) and nascent autophagosomes followed by accumulation of acyl‐CoA synthetase, Faa1, on nucleated phagophores to activate the local synthesis of fatty acids, needed for elongation of phagophore (Figure 3a) (Schütter et al., 2020). The long‐chain acyl‐CoA synthases (ACSs) are a host of 6 (yeast) to 13 (mammals) peripheral and transmembrane proteins (Schütter et al., 2020). These enzymes cause thio‐esterification of free FAs with coenzyme A (CoA) by an ATP hydrolysis‐driven strategy (Figure 4) (Schütter et al., 2020). Within minutes following activation, activated FAs allow for the rapid expansion of membranes of phagophores, leading to the formation of cup‐shaped structures that engulf the cargo and ultimately separate it from the cytosol by complete encapsulation and formation of a double‐membrane‐bound structure the so‐called autophagosome. The outer membranes of these autophagosomes, then, fuse with lysosomes and the inner‐membrane‐bound cargo, the so‐called, autophagic body, is digested by native lysosomal hydrolases, releasing nutrients needed by cells (Figure 4). Based on such an intimate relationship, it has been suggested that autophagic membranes are derived from ER or that, alternatively, such contacts might represent the initial stages of ERphagy (Bento et al., 2016).

Another alternative source for the autophagosomal membrane is derived from mitochondrial membranes. This view is supported by the fact, that during starvation, ATG5 transiently appears in the outer membrane of mitochondria, followed by the appearance of LC3. Others suggest that in mammalian cells, autophagosomes are formed at the ER‐mitochondrial interface, since disrupting such interactions impairs the starvation induced autophagy (Bento et al., 2016). As shown by the rapid integration of labeled plasma membrane lipids into phagophores and autophagosome, another source of autophagosomal membranes is thought to be the plasma membrane. Given that the same set of lipids and proteins participate in the generation of both phagosomes and endosomes, it is thought that both of these processes follow similar membrane‐trafficking processes. This view is supported by the fact that loss of VPS34 adversely impacts both the biogenesis of endosomes and phagosomes and that the master regulator of endocytosis, the GTPase, RAB5 also coordinates autophagy (Bento et al., 2016). Another prevailing idea is that ER‐Golgi Intermediate Compartment (ERGIC) is the source of phagosomal membranes.

## 4 THE CORE PATHWAYS IN AUTOPHAGY

The canonical inducers of autophagy are engaged by stress or physical exercise. The core process of autophagy is initiated either by inhibition of the mechanistic target of rapamycin (mTOR) or activation of 5′ AMP‐activated protein kinase (AMPK) (Figure 4). The biogenesis of lysosomes is also coupled to the activity of both mTOR (via phosphorylation) and AMPK via folliculin (FLCN). AMPK is activated when the nutrient levels fall or when the AMP/ATP ratio rises, and this, in turn, activates the ULK1 (Figure 4) (Rubinsztein et al., 2011).

mTOR is comprised of mTOR complex 1 and mTOR complex 2 (mTORC1 and 2). mTORC1 participates in many actions that require energy, and it increases cellular biomass, drives cell growth, and regulates the autophagic recycling of cell components under nutrient deprivation. mTOR is significant to the activation of autophagy and for the recruitment of autophagy proteins (Atg in yeast and ATG in humans) that are essential for various aspects of autophagy. mTOR also blocks autophagy and controls the lysosomal degradation (Figure 4) (Deleyto‐Seldas and Efeyan, 2021). In cases, that mTOR is inhibited, autophagic recycling of cellular components reactivates mTORC1, providing a direct link and complex regulatory loops between mTOR activation and autophagy, where autophagy lies both upstream and downstream from mTOR (Deleyto‐Seldas and Efeyan, 2021).

Most Atg proteins are cytosolic and transiently associate with the PAS by interacting with other Atg proteins. For example, Atg2‐18 complex, which is conserved in yeast to mammals, physically tethers phagophores to the neighboring ER, organizing ER‐phagophore membrane contact sites (MCSs) with Atg2 mediating phospholipid transport between opposing membranes (Figure 3a) (Schütter et al., 2020). On the contrary, the yeast Atg9, and its ubiquitous mammalian homolog, ATG9A and the ATG9B homologs which are found only in the pituitary gland and placenta, are required for the elongation of phagophore membranes and for the coordination of membrane transport from donor sources to the phagophore (Mari et al., 2010, Schütter et al., 2020).

In the presence of nutrients, the mTOR stimulates growth and prevents cell death. However, upon nutrient deprivation or stress, the mTOR is inhibited by four complexes. These include unc‐51‐like kinase (ULK) complex, the PI3K complex, transmembrane protein complexes, and ubiquitin‐like protein conjugation systems (Figure 4) (Boya et al., 2013). The unc‐51‐like kinase (ULK) complex is made of a tetrameric complex made of ULK‐1, ATG13, ATG101, and FAK‐family interacting protein (FIP200) (Figure 4). Initially, the assembled ULK complexes recruit and phosphorylate the phosphatidylinositol 3‐kinase (PIK3C3, VPS34)‐regulated autophagy protein 1 (AMBRA1), which in turn, activates the P13K complex known to be required in different forms of autophagy (Noda and Inagaki, 2015). In mammalian cells, the class III PI3K participates in various membrane‐trafficking events, whereas the PI3K and Beclin 1 primarily mediate membrane nucleation. The ATG14 complex, which functions in a manner similar to the yeast PtdIns3K complex I, is comprised of homologs of Vps34 (PIK3C3)‐AMBRA1, and PI3K regulatory subunit 4 (PIK3R4, VPS15) and is activated by the binding of Vps30 (Beclin 1; BECN1) to Vps34 (PIK3C3)‐AMBRA1 and is inhibited by BCL2 (Figure 4). The transmembrane protein complexes include Atg9 and WIPI whereas the ubiquitin‐like protein conjugation systems are made of Atg12 and LC3 (Boya et al., 2013).

In order to prevent the premature fusion of vesicles with lysosomes, in yeast, the Atg5‐Atg12‐Atg16 complexes are recruited to PAS where they associate with the outer membrane of the phagophores. These complexes are then dissociated and released upon formation of the autophagosomes. The ubiquitin‐like protein conjugation systems stimulate the binding of phosphatidylethanolamine (PE) and the Atg8/microtubule‐associated protein 1 light chain 3 (LC3) which binds the lysosome with a high affinity forming the LAP‐engaged phagosome (LAPosome). LC3 participates in phagophore elongation, whereas the GABA type A receptor‐associated proteins (GABARAP) contribute in the later stages of autophagic maturation. In yeast, the Atg4, Atg7, and Atg3 process LC3 into LC3‐II, a molecular marker for autophagosomes, which is present both in the outer and inner lysosomal membranes (Figure 4). In mammals, the homologs of yeast Atg8 are comprised of LC3 and GABARAP (Feng et al., 2014). The proLC3 is cleaved after the C‐terminal glycine to form cytosolic LC3I. LC3I is then conjugated to PE to generate the membrane‐associated LC3‐II form (Feng et al., 2014). The GABARAP proteins also experience a similar post‐translational modification. Some types of autophagy are driven by a ligand‐receptor scaffold system. For example, in yeast, the mitochondrial membrane Atg32 acts as a receptor for mitophagy whereas Atg36 acts as a receptor for pexophagy. These receptors bind to the Atg11 which interacts with Atg8 to link the organelle to the autophagic machinery (Feng et al., 2014).

## 5 AUTOPHAGY MAINTAINS HOMEOSTASIS, AND RESTRAINS STRESS, INFLAMMATION, AND TUMORIGENESIS: THE GOOD

Autophagy is a conserved mechanism, necessary for homeostasis, for relief from stress and for prevention of disease (Shintani and Klionsky, 2004). Targeted knockout of Atg related genes causes embryonic lethality at 4–8 cell stage, neurodegeneration, axonal degeneration, hepatomegaly and hepatic failure, cardiac hypertrophy and dysfunction, and disorganized mitochondria, atrophy of fast muscle fibers, muscle atrophy, decreased white adipose tissue, impaired β cell mass and function in pancreas, decreased T and B cells and lymphopenia, severe anemia, late onset glomerulosclerosis, and decreased amino acid levels, features that are commonly found during aging (Mizushima & Levine 2010). Tissue‐specific knockouts of autophagy genes lead to features observed in senescent cells such as the accumulation of p62, lipofuscin, oxidized proteins that undergo carbonylation, carboxymethylation, or nitrosylation, ubiquitin and intracellular inclusion bodies that contain ubiquitinylated proteins (Rubinsztein et al., 2011). Autophagy is vital to blocking the untimely removal of cells by necrosis and apoptosis, likely due to the release of Bcl‐2 from Beclin‐1 and FLIP from Atg3 for blocking the intrinsic and extrinsic apoptosis pathways and also by removal of damaged mitochondria to prevent the unwarranted apoptosis caused by mitochondrial membrane permeabilization (MMP) (Kroemer et al. 2007).

In adults, under basal condition, autophagy regulates the intracellular levels of nutrients, and the availability of metabolites, including amino acids, lipids (lipophagy), carbohydrates (glycophagy), RNA (RNAautophagy), and DNA (DNAautophagy) (Figure 1). In various conditions, particularly during stress and nutritional deficiency, autophagy leads to the clearance of the glycogen whereas impairment of autophagy leads to age‐related accumulation of glycogen (Aman et al., 2021). Genetic deficiency in the lysosomal hydrolytic enzyme, acid α‐glucosidase (GAA), leads to Pompe disease, a rare, progressive, and often fatal muscular disease characterized by the accumulation of glycogen storage in cardiac, smooth and skeletal muscles (Aman et al., 2021). Lipid homeostasis is maintained by lipolysis which involves the hydrolysis of triglycerides in lipid droplets into fatty acids by specific lysosomal acid lipases and by lipophagy (Singh et al., 2009). Autophagy also targets RNA and DNA by lysosomal degradation through LC3‐dependent autophagic degradation of stress granules (Aman et al., 2021). Lysosomal membrane protein, LAMP2C has been shown to directly bind to RNA or DNA before it is degraded within lysosomes (Aman et al., 2021). Moreover, the lysosomal RNA/DNA transporter, SID1 transmembrane family, member 2 (SIDT2), is considered to mediate direct uptake of RNA and DNA for lysosomal degradation (Aman et al., 2021). Autophagy also engages both p62‐ and NDP52‐dependent degradation of retrotransposon RNA (Aman et al., 2021). Together, the available data suggest that loss of autophagy contributes to the accumulation of damaged or unnecessary molecules, promotes inflammation, cancer, and even accelerates aging (Aman et al., 2021).

A key function of autophagy is the cytoplasmic turnover of organelles (ERphagy, mitophagy, lysophagy, and nucleophagy) through selective membrane‐bound and soluble autophagy receptors (Figure 1). The best studied among these, is mitophagy and it has been demonstrated that highly depolarized mitochondria are removed by Ser65‐phosphorylated ubiquitin of the soluble selective autophagy receptors NDP52, optineurin and p62, through PINK1‐ and parkin‐mediated pathways (Pickles et al., 2018).

ATG5 deletion has confirmed its role in protein and organelle turnover, and in the maintenance of homeostasis in different tissues including salivary gland and adipose tissues. ATG5 is involved in the development of B lymphocytes, as a guardian of immune integrity, in kertain K5 expression in epithelia. This autophagy gene is also important for the prevention of osteoarthritis, cataract, dextran sodium sulfate‐induced colitis and autophagic cell death and in the elimination of mitochondria from embryonic reticulocytes (Morgan‐Bathke et al., 2013, Honda et al., 2014, Baerga et al., 2009, Sukseree et al., 2013, Ye et al., 2018, Mizushima et al., 2011, Miller et al., 2008, Pyo et al., 2005, Nishino et al., 2021). Deletion of Atg7 has shown a role for this autophagy gene in adipogenesis, hyperglycemia, glucose intolerance, and hypo‐insulinemia, disorders that are common in aging (Bouderlique et al., 2016, Zhang et al., 2009, Jung et al., 2008, Ebato et al., 2008). Targeted deletion of both Atg5 and 7 leads to mild growth retardation, caspase dependent cell death of chondrocytes, retinal degeneration, and acute myeloid leukemia (Vuppalapati et al. 2015, Takamura et al., 2011, Liu et al., 2016, Zhang et al., 2017).

Autophagy is required for adaptation to and alleviation of cellular stress. Autophagy response to stress includes selective degradation of LC3/GABARAP‐binding proteins. Autophagy regulates lipid metabolism during metabolic stress and starvation by degradation of the nuclear receptor co‐repressor 1 (NCoR1), alleviates oxidative stress through NRF2 antioxidant pathway by degrading KEAP1, maintains homeostasis in islet cells in response to high fat diet, synchronizes the circadian rhythms by the degradation of CRY1, regulates the immune response and the autophagic degradation of retroviral particles by LC3‐binding protein, TRIM5α, suppresses p53 induction and cell death in neurons, and relieves ER and nutrient stress (Altman et al., 2011, Ferraro and Cecconi 2007, Quan et al., 2012, Kroemer et al., 2010).

AMP‐activated protein kinase plays a central role in energy and metabolism by sensing the energy level of cell by the AMP to ATP ratio, and integrates several stress responses with the initiation of autophagy (Kroemer et al., 2010) AMPK participates in a positive amplification loop with Sirt1 and is activated by phosphorylation of a threonine residue on its catalytic α subunit by a host of upstream kinases (Kroemer et al., 2010). These kinases include the liver kinase B1 (LKB1), which is activated by energy depletion, the calcium/calmodulin kinase kinase‐β (CaMKKß), which is activated by the level of the cytosolic Ca^2+^, and TGFß‐activated kinase‐1 (TAK‐1), that causes IKK activation (Ruderman et al., 2010). AMPK induces autophagy by inhibiting mTORC1 through induction of phosphorylation of tuberous sclerosis complex 2 (TSC2) and by phosphorylating Raptor at Ser863, the regulatory associated protein of mTOR and may even directly impacts autophagy by interacting with ULK1 (Kroemer et al., 2010) (Figure 4). mTOR is also a conserved signaling pathway that senses intracellular and extracellular levels of nutrients, and growth factors with mTOR complexes (mTORC) 1 and 2 regulating cellular energy (Linke et al., 2017). mTOR is inhibited by AMPK and by the nutrient deprivation (Jung et al., 2010). mTOR1 is considered to act on the most upstream stage of autophagy pathway by regulating Atg1, an evolutionarily conserved serine/threonine kinase required in nucleation, the early event for initiation of membrane as well as PAS (Jung et al., 2010). TOR pathway regulates the localization and activity of ULK by phosphorylation of ULK complex and interaction of Atg1 with Atg13, Atg17, Atg29, and Atg31, for instance, by reducing the affinity of Atg1 for Atg13 by phosphorylating Atg13 at multiple residues (Jung et al., 2010) (Figure 4).

Autophagy prevents the activation of inflammasome, removes and degrades inflammasome inducers from cytosol, sequesters the pro‐inflammatory cytokines such as IL1β and limits inflammation induced pathologies and infection (Harris et al., 2017, Matsuzawa‐Ishimoto et al., 2018). Autophagy proteins activate RIG‐I signaling and the NLRP3 inflammasome and protect against the inflammation induced by excessive ROS produced by damaged mitochondria and regulate immune response by autophagy independent mechanisms that involve interaction of autophagy proteins with immune signaling (Levine and Kroemer, 2019). Autophagy also prevents sustained innate immune signaling and the cytosolic DNA sensing innate immunity by the cooperative actions of the enzyme, cGAS (cyclic GMP‐AMP synthase) and stimulator of interferon genes (STING) (Levine & Kroemer, 2019). This interaction involves the cGAS‐mediated generation of cGAMPs, which in turn, activates ULK1 that curbs the actions of STING by phosphorylation, and consequently hampering the STING induced cytokine production (Konno et al., 2013). Beclin 1 also binds cGAS leading to halting the cGAMP synthesis and interferon production (Liang et al., 2014). Autophagy is also involved in regulation of both innate and adaptive immunity and in survival of the inflammatory cells, including neutrophils, macrophages, and lymphocytes (Qian et al., 2017). Autophagy also promotes the production of cytokines (IFN‐α, IFN‐β, IFN‐γ, TNF‐α, IL‐1, IL‐2, IL‐4, IL‐6, IL8, IL‐10, IL‐13, IL‐18, TGF‐β, MCP‐1, Th2 cytokines) and chemokines, and enhances killing of pathogens in macrophages by autophagosome dependent and independent mechanisms, enhances phagocytosis by neutrophils and by autophagy dependent cell death. Autophagy is crucial to the T‐ and B‐cell development and survival, T‐cell antigen presentation and B‐cell differentiation (Qian et al., 2017). Autophagy is instrumental in the protection against pathogens such as *Rickettsia conorii*, *Listeria monocytogenes*, *Streptococcus pyogenes,* and *Mycobacterium tuberculosis* (xenophagy) (Aman et al., 2021).

One of the key features of autophagy‐related genes is their role in preventing tumorigenesis, and their suppression has been shown to promote cancer cell growth (White 2015). For example, Beclin1 is considered to be a haplo‐insufficient tumor suppressor gene which is monoallelically lost in 40%–75% of human prostate, breast, and ovarian cancers (White 2015). Further evidence is provided by showing that inhibition of autophagy enhances tumor growth. Furthermore, inactivation of Beclin 1 and Atg5 increases the incidence of cancer in mice, monoallelic deletion of Beclin 1 promotes tumorigenesis, and the induction of autophagy by over‐expression of Beclin 1 inhibits tumor progression (Liang et al., 1999, Qu et al., 2003, Levine 2007). The loss of autophagy‐related genes has also been shown to lead to carcinogen induced fibrosarcoma, and loss of Atg5 and Atg7, which reduces autophagy, is associated with a high incidence of liver tumors (Takamura et al., 2011, Mizushima and Levine 2008, Kimmelman and White 2017, Levine 2007).

## 6 ROLE OF AUTOPHAGY IN AGING: THE BAD

Unfortunately, the protection afforded by autophagy is progressively erased with age. For instance, Atg5, Atg7, and Beclin 1 are down‐regulated in the normal aging brain, whereas, in osteoarthritis, the levels of ULK1, Beclin 1, and LC3 fall (Rubinsztein et al., 2011). In hepatocytes of aged rats, alongside the increase in cytosolic Hsc70, and coordinate with decreased binding and lysosomal uptake of cargo, there is a significant rise in degradation and hence reduced half‐life levels of LAMP2A in lysosomal membranes (Cuervo and Dice, 2000, Kiffin et al., 2007). Indeed, under basal conditions, the half‐life of LAMP2A in early passage fibroblasts is reduced from 38 to 26 h once the senescence settles (Kiffin et al., 2007).

The reduced level of autophagy is consequential at any age, since young mice with conditional tissue‐specific knockouts of Atg7 or Atg5 experience profiles similar to those found in aging, which include accumulation of disordered and defective mitochondria, increase in lipid droplets, increase in age pigment, lipofuscin, increase in protein oxidation, reduced differentiation of myogenic progenitors into muscle or fat, neurodegeneration, and sarcopenia (Rubinsztein et al., 2011). Acute deletion of Atg7 in mice leads to the dedifferentiation and loss of the valuable brown adipose tissue (BAT), and to the reduction of BAT markers such as mitochondrial uncoupling protein 1 (UCP1), features that are also induced during aging (Martinez‐Lopez et al., 2013, Zoico et al., 2019).

Loss of macroautophagy hampers cell cycle progression and induces aneuploidy, likely due to the loss of genome integrity (Matsui et al., 2013). The decrease in lysosomal proteolytic activity and reduced autophagic flux appear to underlie the age‐related diseases in *C. elegans* to rodents and humans (Leidal et al., 2018).

Others adhere to the opinion that some of the side effects of reduced autophagy in aging originate from loss of quality control in mitophagy that prevents the removal of damaged mitochondria, causing a progressive mitochondrial uncoupling, reduced oxidative phosphorylation and energy production, and an increase in reactive oxygen species (ROS) (Vijg and Campisi, 2008). Loss of Atg7 is associated with defects in mitochondrial respiration, resting mitochondrial oxygen consumption, and a compensatory rise in glycolysis in muscle cells (Wu et al., 2009). Similarly, in pancreatic β cells, loss of Atg7 causes mitochondrial dysfunction and elevated levels of oxidative stress (Wu et al., 2009). Parkin and PINK1, the “eat‐me” signals are required for mitophagy, that is initiated by PINK1 mitochondrial localization sequence and PINK1 kinase activity. Both events are required for the translocation of E3 ligase Parkin to the depolarized mitochondria. This is followed by the formation of two distinct poly‐ubiquitin chains, linked through Lys 63 and Lys 27 (Geisler et al., 2010). Consistent with such a role, mutations in Parkin interfere with mitochondrial translocation, ubiquitination, and final clearance of mitochondria by mitophagy.

Together, these data show that autophagy is required for prevention of aging and that maintenance of autophagy and restoration of autophagy genes to their youthful levels can counteract the age‐related damages and declines. In fact, correction of the loss of autophagy in aging reduces the aging disorders, and reduces ER stress while increasing glucose tolerance (Yang et al., 2010). Restitution of LAMP2A to normal levels in aging decreases the polyubiquitinated protein aggregates, oxidized proteins, and apoptosis (Zhang and Cuervo, 2008). Tissue restricted Atg7 over‐expression in the liver is sufficient to correct the autophagic defects that result from suppressed Atg7 expression including ER stress and insulin resistance. These beneficial effects are erased when the Atg5, the downstream mediator of Atg7, is blocked (Yang et al., 2010). On a larger scale, studies in yeasts, worms, flies, and mice have all demonstrated that over‐expression of autophagy‐related genes counteracts aging, as evidenced by the extension of lifespan.

## 7 ROLE OF AUTOPHAGY IN AGING, SENESCENCE, AND TUMORIGENESIS: THE UGLY

Both senescence and an increase in incidence of tumors are key features of aging tissues. Whereas senescent cells experience a high metabolic activity, they remain replicatively incompetent (Hörtensteiner and Feller 2002). This association is replicated in human mammary epithelial cells, by showing that genetic and pharmacologic inhibition of cyclin D1 activity up‐regulates autophagy whereas simultaneous inhibition of Cdk4/6 and autophagy enhances the development of senescence (Brown et al., 2012). Aging fibroblasts and bile duct cells of patients with biliary cirrhosis display features of senescence, including joint increase in LC3 and p62 (Sasaki et al., 2012). Autophagy preceding senescence are also seen in cholangiocytes in fibrosing cholangiopathies, suggesting that autophagy might play a causal role in senescence (Nakanuma et al., 2015, Sasaki et al., 2012). Consistent with this, human fibroblasts that were immortalized with telomerase (hTERT‐BJ1) and stably transfected with autophagy genes (BNIP3, CTSB or ATG16L1), showed features of senescence including mitophagy, cellular hypertrophy, increase in the CDK inhibitor, p21(WAF1/CIP1), β‐galactosidase activity, aerobic glycolysis, and ketone body production (Capparelli et al., 2012). Autophagy is also observed in cancer‐associated fibroblasts (CAF) that experience senescence (Capparelli et al., 2012). Interestingly, similar to CAF, autophagic‐senescent fibroblasts increase the mitochondrial metabolism in nearby cancer cells (Capparelli et al., 2012).

There is a conundrum in the relationship of autophagy and senescence, since autophagy appears to have both anti and pro‐senescence effects (Kwon et al., 2017). On one hand, the normal level of autophagy appears to restrict and postpone senescence. This is supported by the fact that interference in autophagy by genetic or pharmacologic means can suppress and postpone senescence, whereas specific depletion of Atg7, Atg12, or LAMP2 induces senescence in human fibroblasts (Gewirtz, 2013, Kang et al., 2011, Young et al., 2009). On the contrary, autophagy might be an effector mechanism for induction of senescence, since some autophagy genes are increased and the autophagy is activated in senescent cells (Young et al., 2009). The development of oncogene‐induced senescence (OIS) also requires the up‐regulation of autophagy which, in turn, supports the development of senescence by regulation by p38a signaling and mTORC2 regulation (Slobodnyuk et al., 2019, Bernard et al., 2020). Over‐expression of the autophagy gene, ULK3 which induces autophagy, also renders cells senescent (Young et al., 2009).

Notably, the senescence‐induced autophagy appears to be dys‐functional. For example, the accumulation of lysosomal SA‐β Gal, a hallmark of senescence, increase in lysosomal permeability, reduced lysosomal acidity, along with an increase in cytosolic acidification, all show that the actions of lysosomes are disturbed in the senescent state (Wiley and Campisi, 2021). Another evidence for the faulty activity of autophagy in senescent cells is degradation of the mammalian SIRT1 protein via the autophagy protein LC3 which causes SIRT1 levels to dwindle in senescent cells, aging tissues in mice and aging human donor tissues such as spleen, thymus, hematopoietic stem, and progenitor cells, as well as in CD8^+^/CD28^−^ T cells (Xu et al., 2020).

Under normal conditions, autophagy restrains senescence and SASP. This is supported by the evidence that inhibition of autophagy delays both HRAS^G12V^ induced senescence and the associated SASP (Kang and Elledge, 2016, Young et al., 2009). However, paradoxically, the autophagy induces senescence, that may be rooted in age induced defects in mTOR since enhanced mTORC1 activity drives characteristic phenotypes of senescence. It appears that depolarization of senescent cell plasma membrane, and defects in amino acid and growth factor sensing, induce persistent mTORC1 signaling which is required for the maintenance of a senescent state, since inhibiting this activity can reverse senescence (Cayo et al., 2021, Carroll et al., 2017). In OIS, generation of a high level of recycled amino acids and other metabolites supports the lysosome‐bound mTORC1 in inducing SASP as evidenced by the release of cytokines IL‐6 and IL‐8 (Narita et al., 2011, Carroll et al., 2017). Spatial coupling of mTOR and autophagy leads to the engagement of mTOR complex 1 (mTORC1), and establishment of SASP by providing the free amino acids that are required for the synthesis of SASP components (Narita et al., 2011). The amino acid pool is created in the so‐called “TOR‐Autophagy Spatial Coupling Compartment” (TASCC) (Narita et al., 2011). The participation of mTOR in causing senescence and SASP is counter‐intuitive since in non‐senescent cells, up‐regulation of autophagy inhibits mTORC1 (Carroll et al., 2017).

The increase in IP3 receptor in neurodegenerative diseases such as Alzheimer's disease, might also be rooted in decreased autophagy (Rubinsztein et al., 2011). The decrease in autophagy might also underlie the metabolic aberrations, seen in aging. For example, in a model of diet‐induced obesity, down‐regulation of autophagy and more importantly, the liver‐specific Atg7 result in ER stress and defective insulin signaling in obese mice (Yang et al., 2010). It also appears that aging may cause a “cargo recognition failure,” resulting in the accumulation of damaged mitochondria in Parkinson's disease, one of the most common neurodegenerative movement disorders (Martinez‐Lopez et al., 2015).

The role of autophagy on tumorigenesis and tumor progression is complex. Disruption of macroautophagy by the loss of a single allele of Beclin1/Atg6 (Class III PI3K) increases the incidence of cancers, showing a suppressive role for autophagy that may be mediated by inducing senescence (Nishida et al., 2009, Liu et al., 2014, Huang et al., 2014). On the contrary, fully transformed cancer cells rely on autophagy to meet and relieve the metabolic stress, induced by their microenvironment (Poillet‐Perez & White, 2019, White et al., 2021). Targeting cyclin D‐CDK4/6 increases the survival of patients with estrogen receptor positive breast cancer and in gastric cancer cells, inhibition of CDK4/6 with Palbociclib induces senescence or death, whereas in cells that express pRB and p53, it induces autophagy (Brown et al., 2012, Valenzuela et al., 2017). Interestingly, the inhibition of this autophagy improves the efficacy of CDK4/6 inhibitors in breast and other solid cancers that exhibit an intact G1/S checkpoint (Vijayaraghavan et al., 2017).

Cancer‐associated fibroblasts (CAFs), the most abundant cells in cancer, display both autophagy and senescence, as evidenced by a secretome that overlaps with that seen in senescent fibroblasts (Sahai et al., 2020, Yan et al., 2019). This combination of autophagy and senescence is consequential, since these cells are known to be critically important in cancer progression and metastasis (Karagiannis, et al. 2012, Chen and Song 2019, Yan et al., 2019). In support of this cooperative interaction, the ATG16L1 fibroblasts that show autophagy, and produce large amounts of ketone bodies (3‐hydroxy‐butyrate), increase the rate of metastasis by up to 11‐fold (Capparelli et al., 2012). Cancer and senescent cells also show common features that include overlapping signaling pathways, involving participation of tumor protein p53 (TP53), cyclin‐dependent kinase inhibitor 1A (CDKN1A/p21), ataxia–telangiectasia mutated (ATM), dephosphorylation of retinoblastoma (RB), and ROS (Rajendran et al., 2019). Another key feature common to the cancer cells, CAF, and senescent cells is their metabolic reprogramming due to their high metabolic demand (Hörtensteiner and Feller 2002, Cairns et al., 2011, Avagliano et al., 2018). Interestingly, autophagy and metabolic reprogramming are also features of stem cells that rely more heavily on glycolysis, rather than oxidative phosphorylation, to meet their ATP demands, perhaps due to their residence in hypoxic niches (Shyh‐Chang, and Ng 2017). For this reason, it is not surprising to find that autophagy is also required for cancer stem cells (CSCs) that exhibit the potential for self‐cell renewal, tumor metastasis and resistance to chemotherapy, and live in microenvironments that are hypoxic, acidic and offer little nutrition (Pattabiraman and Weinberg, 2014). These microenvironmental cues are significant in the engagement of autophagy, since in nutrient‐poor conditions, autophagy is greatly increased (Kuma et al., 2010). Similarly, moderate hypoxia (~0.1–3%) rapidly increases HIF‐1 which sustains cell survival by promoting mitophagy, whereas severe hypoxia (<0.1%) acts through HIF‐1‐independent mechanisms that involve AMPK, mTOR, and unfolded protein response (UPR) with the potential to cause cells to undergo autophagic cell death (Mazure and Pouysségur, 2010). The importance of autophagy in tumorigenesis has recently come into focus by the fact, that knockdown of *ATG* genes in xenografts of breast CSCs hampers their self‐renewal and growth in mice (Boya et al., 2018). Together, these results show that senescent cells, CAF, stem cells, and cancer cells exhibit common features that appear to result from their metabolic demands driven by microenvironmental conditions that support autophagy.

Another consequence of reduced autophagy in aging is the shortening of lifespan. For example, in *Saccharomyces cerevisiae* (*S. cerevisiae*) reducing macroautophagy shortens lifespan and among the 117 short‐lived mutants, a microarray screen showed 10, to have mutations in Atg (Matecic et al., 2010, Rubinsztein et al., 2011). In *C. elegans*, loss of function of Atg1 (Unc‐51), Atg7, Atg18, and Beclin‐1 and in *D. melanogaster*, induction of reduced expression of Atg1 and Atg8 are associated with a short lifespan (Rubinsztein et al., 2011). The reverse is true when the autophagy is increased. For example, over‐expression of the TFEB orthologue HLH‐30 which increases TFEB‐driven expression of macroautophagy‐related genes increases longevity in 6 distinct longevity models in *C. elegans* (Lapierre et al., 2013). Along the same line, the over‐expression of Atg5 in mice enhances resistance to oxidative stress‐induced cell death, increases leanness, insulin sensitivity, and motor function, and extends lifespan by 17.2% (Pyo et al., 2005).

Tissue‐specific over‐expression of single autophagy genes has been shown to be sufficient for increasing longevity (Hansen et al., 2018). Also, the mTOR inhibitor, rapamycin that reduces protein synthesis and promotes autophagy extends lifespan in organisms as diverse as yeast, nematodes, flies and mice and protects, against the neurodegenerative diseases, such as Alzheimer's disease (Aman et al., 2021). Similarly, the mTOR‐independent enhancement of autophagy through AMPK pathway by metformin and trehalose also extends lifespan and protects against the development of neurodegeneration in experimental animals (Aman et al., 2021). Spermidine that enhances autophagy by inhibition of the EP300 acetyltransferase and resveratrol that activates NAD^+^ dependent increase in deacetylase activity of SIRT1 and mTOR inhibition are also known to extend the lifespan (Tabibzadeh, 2020).

It has been shown that calorie restriction (CR) without causing malnutrition delays the onset of age‐related diseases including diabetes, brain atrophy, cardiovascular disease, and risk of cancer and decreases mortality in rhesus monkeys (Colman et al., 2009). The life extension by CR appears to be dependent on increased autophagy since in yeast and *C. elegans*, the effect of CR on life extension is extinguished by knockout of Atg genes (Rubinsztein et al., 2011). The life extension by calorie restriction (CR) is also due to the increase in autophagy by establishment of a positive feedback loop that engages the activation of AMPK, Sirt1, as well as by TOR inhibition induced by loss of insulin and insulin growth factor (IGF) signaling (Kenyon, 2010). In fact, the suppression of insulin/IGF‐1 pathway by RNAi knockdown or mutations of *Daf‐2* that cause loss of function, and insulin/IGF‐1 pathway mutants, IRE‐1 and XBP‐1 have all shown to enhance resistance to ER stress and to extend the lifespan (Henis‐Korenblit et al., 2010). These beneficial effects can be overcome by the loss of FOXO family member, *Daf‐16* which is activated in the mutants. The resistance to ER stress and life extension in Daf‐2 mutants appear to require both Daf‐16 activation and XBP‐1 (Henis‐Korenblit et al., 2010). Similar studies in mammals have shown that FOXO3a is required for autophagy (Rubinsztein et al., 2011). The impact of Atg in life extension also requires the participation of sirtuin 1 (SIRT1) and its orthologs, Sir2 in yeast and sir‐2.1 in *C. elegans* since loss of Atg genes prevents the life extension induced by Sir2 over‐expression (Rubinsztein et al., 2011).

## 8 CONCLUDING REMARKS

**FIGURE 5:**
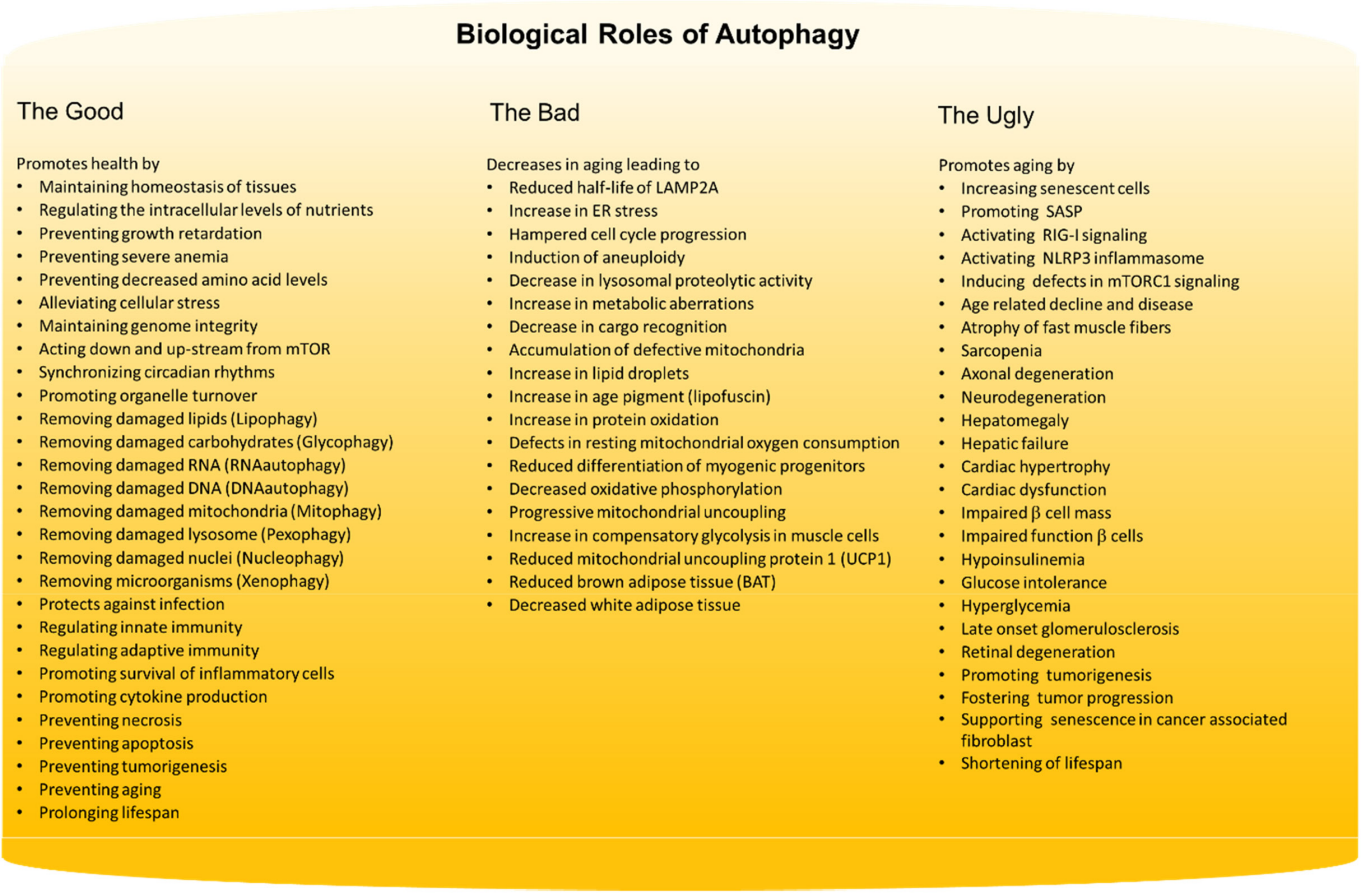
Good, the bad, and the ugly aspects of autophagy in normal homeostasis, and in pathologic conditions that promote aging, senescence, and tumor progression

There is a need to further understand the role of autophagy in aging since, although early in life in *C. elegans*, suppression of autophagy adversely impacts longevity, the knockdown of a specific subset of autophagy genes in mature adults may have an opposite effect (Aman et al., 2021). It has also been shown that whereas a mild increase in autophagy by over‐expressing the major autophagy kinase, Atg1, extends lifespan in *Drosophila melanogaster*, a strong increase in autophagy shortens their lifespan suggesting that, depending on the age, autophagy might be required to be finetuned (Bjedov et al., 2020). Excess autophagy appears to be detrimental to life by inducing atrophy or generalized loss of cells. Also, despite the beneficial effects of autophagy, significant increase in general autophagy and decline in mitophagy are observed in premature aging and progeria such as ataxia telangiectasia, xeroderma pigmentosum group A, and Cockayne syndrome (Aman et al., 2021). These findings suggest that decline in mitophagy rather than macroautophagy may be contributing to the progeria. Together, it appears that autophagy is age and context dependent and its activation relies heavily on extra‐ and intracellular cues, driven particularly, by cellular metabolic demands and microenvironmental conditions (Figure 5).

## Data Availability

Data sharing is not applicable to this article as no new data were created or analyzed in this study.
